# High-Density Expression of Ca^2+^-Permeable ASIC1a Channels in NG2 Glia of Rat Hippocampus

**DOI:** 10.1371/journal.pone.0012665

**Published:** 2010-09-10

**Authors:** Yen-Chu Lin, Yu-Chao Liu, Yu-Yin Huang, Cheng-Chang Lien

**Affiliations:** 1 Institute of Neuroscience and Brain Research Center, National Yang-Ming University, Taipei, Taiwan; 2 Department of Anesthesiology, Cheng Hsin General Hospital, Taipei, Taiwan; University of California, Berkeley, United States of America

## Abstract

NG2 cells, a fourth type of glial cell in the mammalian CNS, undergo reactive changes in response to a wide variety of brain insults. Recent studies have demonstrated that neuronally expressed acid-sensing ion channels (ASICs) are implicated in various neurological disorders including brain ischemia and seizures. Acidosis is a common feature of acute neurological conditions. It is postulated that a drop in pH may be the link between the pathological process and activation of NG2 cells. Such postulate immediately prompts the following questions: Do NG2 cells express ASICs? If so, what are their functional properties and subunit composition? Here, using a combination of electrophysiology, Ca^2+^ imaging and immunocytochemistry, we present evidence to demonstrate that NG2 cells of the rat hippocampus express high density of Ca^2+^-permeable ASIC1a channels compared with several types of hippocampal neurons. First, nucleated patch recordings from NG2 cells revealed high density of proton-activated currents. The magnitude of proton-activated current was pH dependent, with a pH for half-maximal activation of 6.3. Second, the current-voltage relationship showed a reversal close to the equilibrium potential for Na^+^. Third, psalmotoxin 1, a blocker specific for the ASIC1a channel, largely inhibited proton-activated currents. Fourth, Ca^2+^ imaging showed that activation of proton-activated channels led to an increase of [Ca^2+^]_i_. Finally, immunocytochemistry showed co-localization of ASIC1a and NG2 proteins in the hippocampus. Thus the acid chemosensor, the ASIC1a channel, may serve for inducing membrane depolarization and Ca^2+^ influx, thereby playing a crucial role in the NG2 cell response to injury following ischemia.

## Introduction

NG2 cells, also known as polydendrocytes, represent a fourth major glial cell population in the mammalian CNS. They express a specific chondroitin sulfate proteoglycan NG2 on the surface, and are morphologically, antigenically and functionally distinct from mature astrocytes, oligodendrocytes and microglia (for review, see [1]). Unlike other types of glial cells, NG2 cells have several neuron-like properties. First, they receive both glutamatergic and GABAergic synaptic inputs [2], [3]. Second, they express Ca^2+^-permeable α-amino-3-hydroxy-5-methyl-4-isoxazolepropionic acid receptors (AMPARs) [4], [5] and display long-term potentiation-like synaptic plasticity [5]. Third, they express the synaptic vesicle protein synaptophysin [6]. Finally, they express voltage-dependent Na^+^ channels and generate graded spikes or action potentials [5], [7], [8].

NG2 cells play an important role in myelination. NG2 cells are capable of differentiating into myelinating oligodendrocytes during the brain development and adulthood ([9], [10]; for review see [1]). It is generally believed that NG2 cells act as a reservoir of new oligodendrocytes throughout life. Intriguingly, it remains unclear about the function of the newly myelinated axons during normal adulthood [9]. Another puzzle is whether NG2 cells are multipotent progenitor cells in the adult brain. Although studies demonstrated that NG2 glia can differentiate into oligodendrocytes, neurons and astrocytes [9]–[12], genetic fate mapping studies did not yet give rise to consistent conclusions about the neuronal and astrocytic fates of NG2 cells *in vivo* (see [1] for the review).

Although whether NG2 cells are multipotent progenitor cells remains debated, they are believed to play an important role in CNS development, repair, and regeneration. Indeed, NG2 cells undergo reactive changes in response to a wide variety of insults to the CNS [3], [13]–[15]. After cortical ischemia, brain-derived neurotrophic factor (BDNF) expression in NG2 cells is markedly elevated [3]. In rats of kainate-induced chronic seizures, NG2 cells not only increase the number of filopodia-like processes but also proliferate in response to pathological hyperactivity [15], [16]. Such an ability to sense environmental changes or pressures requires the appropriate expression and localization of many proteins, indicating that NG2 cells have made a significant investment in detecting aversive stimuli. More recently, the neuronally expressed, proton-gated acid-sensing ion channels (ASICs) are shown to play crucial roles in cerebral ischemia, epilepsy and many neurological disorders [17]–[20] (for review, see [21]). Given that acidosis is a common feature of brain in acute neurological injury, particularly in ischemia and epilepsy, we thus hypothesize that a drop in pH may be the linkage between the pathological process and activation of NG2 cells. Such a postulate raises the question of whether NG2 cells express the acid chemsensor ASIC and if so, what are their functional properties and subunit composition?

To answer these questions, we combined electrophysiology, Ca^2+^ imaging and immunocytochemistry to investigate the functional expression of ASICs in defined NG2 cells in the CA1 area of the hippocampus. Our results show that NG2 cells express high density of ASIC1a channels. Functional activation of ASIC1a channels leads to marked membrane depolarization and an increase in intracellular Ca^2+^. Thus, ASIC1a channels in NG2 cells may serve to transduce local pH changes into electrical and metabolic/Ca^2+^ signaling under a variety of physiological and pathological conditions.

## Methods

All animal studies were conducted in accordance with principles and procedures outlined in the National Institutes of Health Guide for the Care and Use of Laboratory Animals. All experiments were conducted using a protocol (approval number 970603 to CCL) reviewed and approved by the Institutional Animal Care and Use Committee (IACUC) of National Yang-Ming University.

### Electrophysiology

Hippocampal slices were prepared from 15- to 21-d-old Sprague-Dawley rats as described previously [22]. Slices were sectioned in ice-cold cutting saline containing (in mM) 87 NaCl, 25 NaHCO_3_, 1.25 NaH_2_PO_4_, 2.5 KCl, 10 glucose, 75 sucrose, 0.5 CaCl_2_ and 7 MgCl_2_ and incubated in the oxygenated (95% O_2_/5% CO_2_) cutting saline with 0.5 µM sulforhodamine 101 (SR101) added in a holding chamber at 34°C for 25 min. Slices were then transferred to oxygenated solution lacking SR101 at 34°C for 10 min [23] and finally were stored at room temperature until used. During experiments, slices were placed in a recording chamber and superfused with oxygenated artificial CSF containing (in mM) 125 NaCl, 25 NaHCO_3_, 1.25 NaH_2_PO_4_, 2.5 KCl, 25 glucose, 2 CaCl_2_ and 1 MgCl_2_. Recording electrodes (3–6 MΩ) were pulled from borosilicate glass (Harvard apparatus). Putative NG2 cells were first identified by the round somata (diameter <10 µm) under infrared differential interference contrast (IR-DIC) microscope (Axioskop 2 FS, Zeiss) and by the lack of labeling for the red fluorescent dye SR101 under epifluorescence illumination [23]. Whole-cell recordings were made at 22–24°C using an Axopatch 200B amplifier (Molecular Devices). Fast application of H^+^ on nucleated patches from NG2 cells was performed as described previously [24]. Double-barreled application pipettes were fabricated from theta glass capillaries (2 mm outer diameter, 0.3 mm wall thickness, 0.12 mm septum, Hilgenberg, Malsfeld, Germany), and mounted on a piezoelectric-based solution switching system (Burleigh LSS-3000, EXFO, Ontario, Canada). The time necessary for complete exchange of solution was measured with an open patch pipette by switching between Na^+^-rich and 20% Na^+^-rich solutions. It was 556±5 µs (*n* = 5) by measuring 20–80% rise time of junction potential change. The membrane surface area of the nucleated patch was calculated as described previously by approximating that nucleated patches were ellipsoid [24]. ASIC currents evoked by acid pulses were applied every 60 - 120 s. In some experiments, ASIC currents in NG2 cells were evoked by focal puffs of H^+^ (1.5 to 6 s with ∼6 psi) using a PicoSpritzer III (Parker Instrumentation). Both the piezoelectric device and PicoSpritzer were triggered by an external TTL pulse generated by Digidata 1322A. Data acquisition (filtered at 5 kHz and digitized at 10 kHz) and pulse generation were performed using a Digidata 1322A and pClamp 9.2 software (Molecular Devices).

### Calcium imaging

Ca^2+^ imaging was performed using an EM-CCD camera (QuantEM 512SC, Photometrics) mounted on an Olympus upright microscope (BX51WI) as described previously [22]. Ca^2+^ indicator Oregon Green 488 BAPTA-1 (OGB-1, Invitrogen) was included in the internal solution containing (mM) 125 Kgluconate, 4 MgCl_2_, 4 K_2_ATP, 10 Na_2_-phosphocreatine, 10 Hepes and 0.3 Na_3_GTP. After loading, the cells were imaged (10–20 ms exposure time; 10 Hz) with 494-nm excitation through a filter set (dichroic mirror DM505/barrier filter BA510IF, Olympus). Background subtracted Ca^2+^ responses were expressed as relative changes in fluorescence (*ΔF/F*).

### Solutions and drugs

The intracellular solution for recording pipettes contained (in mM): 135 Kgluconate, 20 KCl, 0.1 EGTA, 2 MgCl_2_, 4 Na_2_ATP, 10 Hepes and 0.3 Na_3_GTP; pH adjusted to 7.3 with KOH. For measuring reversal potential, we used Cs-based internal solution containing (in mM): 130 CsMeSO_3_, 10 EGTA, 2 MgCl_2_, 2 Na_2_ATP and 20 Hepes. For measuring voltage-gated Na^+^ currents, we used Cs-based internal solution with addition of 5 mM 4-aminopyridine in the bath. The Hepes-buffered Na^+^-rich external solution in the control barrel contained (mM): 135 NaCl, 2.5 KCl, 0.5 CaCl_2_, 1 MgCl_2_ and 10 Hepes; pH adjusted to 7.4 with *N*-methyl-*d*-glucamine (NMDG). To activate ASIC currents, we applied 2-(*N*-morpholino)ethanesulfonic acid (MES)-buffered Na^+^-rich external solution in the test barrel containing (mM) 135 NaCl, 2.5 KCl, 0.5 CaCl_2_, 1 MgCl_2_ and 10 MES, adjusted to desired pH with NMDG. In whole-cell recording experiments, 2 mM kynurenic acid and 1 µM gabazine (Park Ellisville) were included to block glutamate-receptor (AMPA and *N*-methyl-d-aspartate (NMDA))- and GABA_A_-receptor-mediated synaptic transmission, respectively. In some experiments, amiloride (Tocris Bioscience) and psalmotoxin 1 (PcTX1, Peptide International) were added to the bath to block ASIC channels; For PcTX1, bovine serum albumin (0.1%) was added in the external solution to prevent its absorption to tubing and containers. All other chemicals were from Sigma except where noted.

### Immunocytochemistry

Staining of biocytin-filled SR101-negative (putative NG2) cells with Alexa-594 conjugated streptavidin (Invitrogen, 1∶400) was performed as described previously [22]. NG2-labeling was done with a mouse-anti-NG2 primary (Abcam, 1∶200) and goat-anti-mouse-Alexa 488 secondary antibody (Invitrogen, 1∶500). Slices were fixed after recording in 4% paraformaldehyde (PFA) in 0.1 M PBS overnight. After washing in PBS, slices were permeabilized with 0.3% Triton-X 100 and then blocked in PBS +10% normal goat serum (NGS, 2 h at 4°C). Primary antibody was applied in PBS +0.3% Triton X-100+5% NGS (48 h, 4°C), and secondary antibody was incubated together with streptavidin conjugate in PBS +0.3% Triton X-100+2% NGS for 2 h at 4°C. After wash, slides were coverslipped with mounting medium Vectashield® (Vector Laboratories). For double immunofluorescence studies, rats were deeply anesthetized and transcardially perfused with saline (0.9% NaCl) followed by 4% PFA. Brains were postfixed (2–6 h) in 4% PFA and placed in 30% sucrose (2 days) and sectioned (20–30 µm) with a cryostate (Leica CM1900). Primary antibodies against NG2 (mouse, 1∶200; Abcam), glial fibrillary acidic protein (GFAP) (rabbit, 1∶400; Dako, Denmark) and ASIC1a (rabbit, 20 µg/ml; Alpha Diagnostic International) and secondary antibodies (goat anti-rabbit-Alexa 488, 1∶500 for GFAP or ASIC1a; goat anti-mouse-Alexa 594, 1∶500 for NG2; Invitrogen) were used. Specificity of the ASIC1a antibody was checked by Western blotting showing that the antibody only detected ∼97 kDa ASIC1a protein and immunostaining of hippocampal slices using the antibody pre-absorbed with an excess of exogenous antigens or with the same PBS, in which primary antibodies were omitted. Slices were imaged by two-photon microscopy as described previously [22].

To quantify the degree of co-localization [25], we analyzed the areas where NG2 (red (R)) and GFAP (green (G)) pixels overlapped became yellow (Y) using the built-in co-localization tool of MetaMorph software (version 7.5.2.0; Molecular Devices). The degree of overlapped areas (yellow pixels) were presented by either Y/R or Y/G.

### Data analysis and statistics

Data were analyzed using Clampfit 10.2 (Molecular Devices) and SigmaPlot 10.0 (Systat Software). Traces in the figures were averages of 3–12 sweeps. Concentration-response curves were fitted with the function:



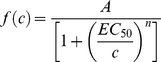
(1)


Where *A* is the constant for the maximal effect, *c* denotes the concentration, *EC*_50_ represents the half-maximal effective concentration, and *n* denotes the Hill coefficient. Data were presented as mean ± SEM. Error bars equal SEM. Statistical significance was tested by the non-parametric Wilcoxon rank-sum or Wilcoxon signed-rank test at the significance level (*P*) as indicated, using Prism 5.0 (GraphPad).

## Results

### Identification of hippocampal NG2-expressing glia

**Figure 1:**
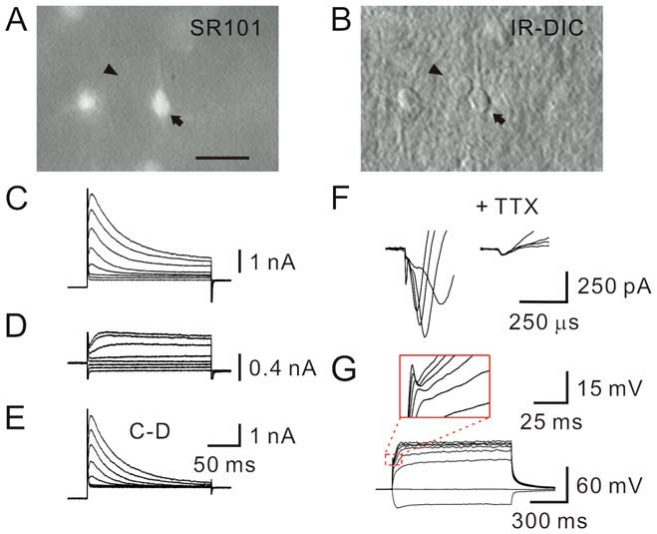
Identification of NG2-expressing cells in the hippocampus. (A) Epifluorescence image showing an SR101(+) cell (arrow) in the *stratum radiatum* of the CA1 area. Arrowhead, an SR101(−) cell, a putative NG2 cell, which was visible under IR-DIC optics in (B). Scale bar, 10 µm. (B) IR-DIC image of (A). (C) Total outward K^+^ currents recorded from an NG2 cell evoked by step voltages (20 mV, 200 ms) from −80 mV to +40 mV with an 1-s prepulse (−120 mV). (D) Delayed-rectifier K^+^ currents evoked in the same cell by the same step voltage protocol in (C) with an 1-s prepulse (−30 mV). (E) A-type K^+^ currents obtained by subtracting (D) from (C). (F) Left, representative inward Na^+^ currents evoked by step voltages (20 mV, 200 ms) from −10 mV to +50 mV with an 1-s prepulse (−120 mV). Right, after addition of TTX (0.5 µM). (G) Voltage responses to the current injection (−100 pA to +600 pA; step, 100 pA).

Glia exhibit electrophysiological diversity predominantly in the developing brain [26], [27]. In addition, the soma shape observed under IR-DIC optics cannot be used to discriminate astrocytes from NG2-glia [27]. However, recent reports showed that the red fluorescent dye SR101 is exclusively taken up by astrocytes after a brief exposure in the intact brain or acute brain slices [23], [28]. We, therefore, made whole-cell recordings from SR101(-) cells (Figs. 1*A* and *B*; see Methods) in the *stratum radiatum* of the CA1 area in acute hippocampal slices from young (postnatal days 15–21) rats. Whole-cell current (*I*)-clamp recordings showed that these putative NG2 cells (*n* = 81) had membrane potential (*V*_m_) of -81.9±0.4 mV and input resistance of 228±15 MΩ. In voltage (*V*)-clamp recordings, they exhibited pronounced outward K^+^ currents, including A-type and delayed-rectifier K^+^ currents during depolarizing pulses (Figs. 1*C*–*E*). In some experiments, after pharmacological blockade of K^+^ channels (see Methods), they displayed TTX-sensitive, voltage-dependent Na^+^ currents (Fig. 1*F*). In *I*-clamp recordings, the majority (86%) of recorded cells displayed a small membrane bump upon strong depolarization (Fig. 1*G*) as described previously [8].

**Figure 2:**
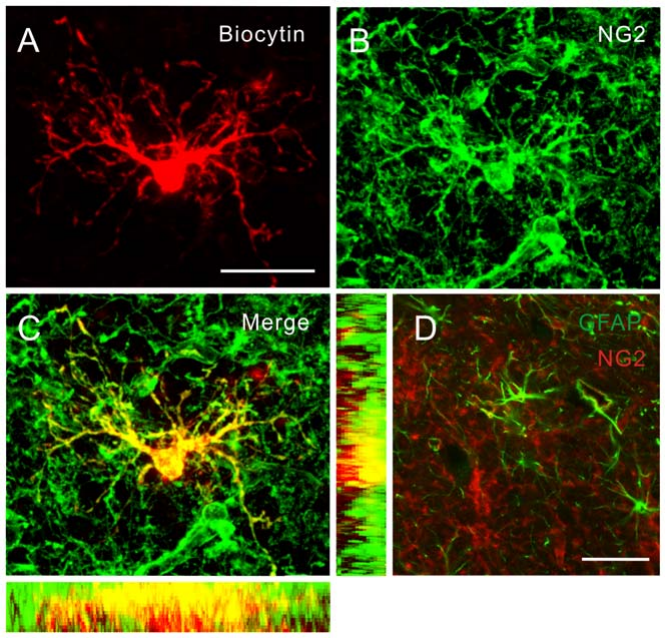
*Post-hoc* morphological and immunocytochemical analysis. (A) Two-photon image stack of a biocytin-filled NG2 cell stained with Alexa-594 conjugated streptavidin. Scale bar, 25 µm. (B) NG2 immunoreactivity of the same slice in (A). (C) Images of (A) and (B) are superimposed. Three orthogonal (*xy*, *xz* and *yz*) planes are shown. (D) Double immunofluorescent labeling of GFAP and NG2 proteins. Scale bar, 25 µm.

During recordings, cells were loaded with biocytin for *post-hoc* histology and immunocytochemistry. *Post-hoc* morphological analyses revealed that the recorded cells (*n* = 42) had the characteristic morphology of NG2-expressing glial cells. They showed a round somata with multiple processes (Fig. 2*A*). All labeled cells (42 cells) were found to be NG2-immunoreactive (Figs. 2*A–C*). We also performed double staining with antibodies to GFAP (green (G)), a marker for astrocytes, and NG2 (red (R)) (Fig. 2*D*). To quantify the degree of co-localization, the overlapped areas (yellow (Y) pixels) were presented by either Y/R or Y/G (see Methods; [25]). These two ratios (Y/G  = 9.8±2.0%; Y/R  = 6.8±0.9%; from 17 sections/2 animals) are similar and below 10%. Overall, double immunostaining of GFAP and NG2 revealed two distinct cell populations (Fig. 2*D*). In addition, unlike our recordings in astrocytes [29], we did not observe any gap junction coupling after single-cell biocytin labeling in all experiments.

### Proton-activated currents are present in NG2 cells

**Figure 3:**
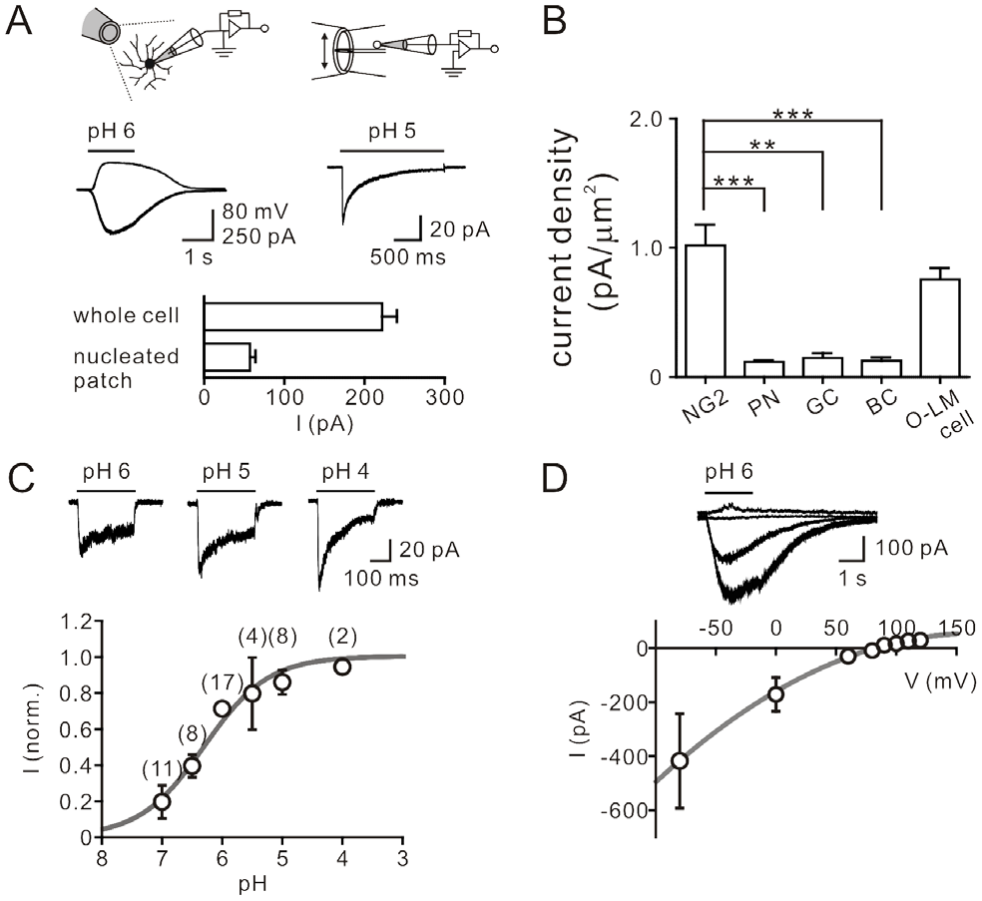
ASIC currents are present in NG2 cells. (A) Top, schematics of whole-cell (left) and nucleated patch (right) recordings. Middle left, puffer application of acid onto an NG2 cell elicited inward currents (*V*-clamp at −80 mV) or membrane depolarization (*I*-clamp, *V*_m_  = −80 mV before the pulse). Middle right, ASIC currents evoked by fast application technique in a nucleated patch from an NG2 cell (*V*-clamp at −60 mV). Bottom, summary of acid-evoked ASIC currents. (B) ASIC current density in NG2 cells compared to various types of neurons in the CA1 area and dentate gyrus. PN, pyramidal neuron; GC, granule cell; BC, basket cell; O-LM cell, *oriens lacunosum-moleculare* cell. **P<0.005; ***P<0.0005. (C) Top, ASIC currents evoked by different pH reductions. Bottom, the normalized peak amplitude of ASIC current plotted against the pH value. Data fit with the Hill equation. (D) Top, overlay of ASIC currents recorded at different potentials (−80, 0, +80 and +100 mV). Bottom, the *I-V* relationship had the reversal potential of +85 mV. Data (pooled from 8 cells) fit with a polynomial function.

We next investigated the functional expression of ASICs in NG2 cells using whole-cell (Fig. 3*A*, left) and nucleated patch (Fig. 3*A*, right) configurations. The later recording configuration was used in most experiments because it unequivocally demonstrated that proton-activated currents were caused by ASIC channels expressed in NG2 cells but not by neighboring cells. Furthermore, it allowed us to examine channel gating under an ideal voltage-clamp condition. Upon focal acid (pH 6) puffing, NG2 cells exhibited pronounced membrane depolarization (40±5 mV, *n* = 19; *I*-clamp *V*_m_  = −80 mV) and transient inward currents (222±19 pA, *n* = 53; *V*-clamp  = −80 mV) (Fig. 3*A*). Similar to whole-cell recordings, nucleated patches from all recorded NG2(+) cells (*V*-clamp  = −60 mV) exhibited transient inward currents (57±7 pA, *n* = 17) in response to the pH change from 7.4 to 5 by fast application technique (Fig. 3*A*, right). Analysis of the transient currents from nucleated patch recordings revealed that the 20–80% rise time and the decay time constant were 2.7±0.2 ms (*n* = 13) and 385±1 ms (*n* = 18), respectively.

Recently we have shown that ASIC current density in the hippocampal neurons is cell type-specific [24]. We thus compared the ASIC current density of nucleated patches from NG2 cells with those from various types of neurons. ASIC current density (1.02±0.16 pA/µm^2^, *n* = 17) in nucleated patches from NG2 cells upon pH 5 application was at least 6-fold larger than those of CA1 pyramidal neurons (PNs, 0.11±0.01 pA/µm^2^, *n* = 27; *P*<0.0005, Wilcoxon rank-sum test; Fig. 3*B*) and dentate gyrus granule cells (GCs, 0.15±0.04 pA/µm^2^, *n* = 5; *P*<0.005, Wilcoxon rank-sum test; Fig. 3*B*). As compared with GABAergic neurons, it was larger than that of basket cells (BCs, 0.12±0.03 pA/µm^2^, *n* = 19; *P*<0.0005, Wilcoxon rank-sum test; Fig. 3*B*), a major type of soma-targeting interneurons, but was comparable to that of *oriens lacunosum-moleculare* (O-LM) cells (0.75±0.08 pA/µm^2^, *n* = 27; *P* = 0.18; Wilcoxon rank-sum test; Fig. 3*B*), a prototype of dendrite-targeting interneurons.

Steep pH dependence, Na^+^ selectivity, and blockade by amiloride are hallmark properties of ASIC gating [17], [30]–[33]. To test the pH dependence, we recorded ASIC currents upon different extracellular pH reductions (Fig. 3*C*). The magnitude of ASIC currents markedly depended on the pH value. The dose-response curve was fitted with the equation (Eq. 1), yielding the pH value of 6.37 for the half-maximal activation and the Hill coefficient of 0.9 (Fig. 3*C*). ASICs are non-voltage-gated Na^+^ channels. We then determined the reversal potential of ASIC currents in NG2 cells by measuring the currents at varied holding potentials (Fig. 3*D*). The *I-V* relationship revealed that ASIC currents reversed at +85 mV, close to the equilibrium potential for Na^+^ (*E*_Na_, +89.5 mV at 23°C) (Fig. 3*D*).

### Pharmacological properties and gating of ASICs in NG2 cells

**Figure 4:**
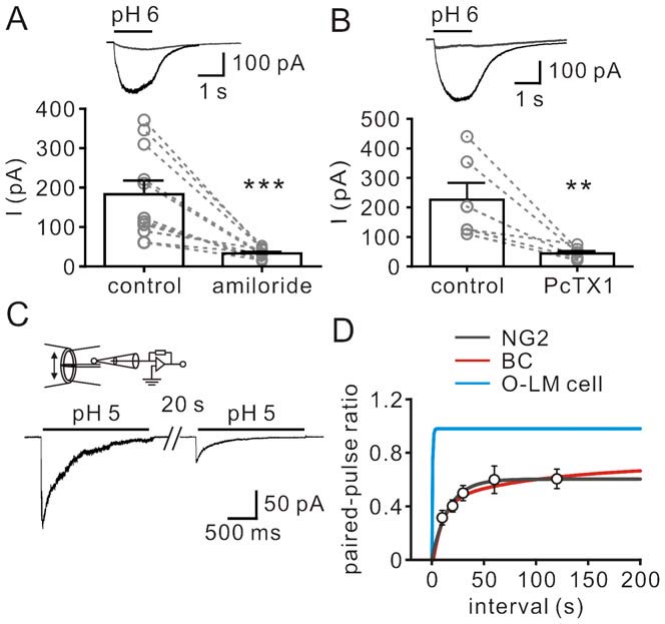
Pharmacological profile and gating of ASICs in NG2 cells. (A) Top, ASIC currents evoked by pH 6 in the control (black) and by co-application of pH 6 and 100 µM amiloride (gray). Traces in the control and in the presence of amiloride were obtained from the same cell. Bottom, summary of the effect of amiloride on ASIC currents. Data obtained from the same cell were connected by dashed lines. ***P<0.0005. (B) Top, ASIC currents evoked in the control (black) and after bath addition of PcTX1 (gray). Bottom, summary of the effect of PcTX1 on ASIC currents. **P<0.005. (C) Representative ASIC currents in a nucleated patch (*V*-clamped at −60 mV) evoked by double pulses (2 s, pH 5) separated by 20 s. (D) Recovery from desensitization of ASIC currents. The black curve represents a single exponential function fitted to the data points (pooled from at least 5 cells). ASIC recovery curves from BCs (red) and O-LM cells (blue) (reproduced from [24]).

Pharmacologically, ASIC currents in NG2 cells were sensitive to amiloride (100 µM), a blocker for the degenerin/epithelial Na^+^ channel (DEG/ENaC) superfamily (control, 183±34 pA; amiloride, 33±4 pA, *n* = 11; *P*<0.0005, Wilcoxon signed-rank test; Fig. 4*A*). Tarantula venom toxin PcTX1 is known to specifically block homomeric ASIC1a channels [34], [35]. We further determined the molecular identity of ASIC channels by examining the sensitivity of ASICs to PcTX1. As shown in Fig. 4*B*, bath application of PcTX1 (30 nM) inhibited 76±7% of the current (*n* = 6; *P*<0.005, Wilcoxon signed-rank test), indicating that homomeric ASIC1a channels compose a large fraction of functional ASICs in NG2 cells.

Homomeric ASIC1a channels are featured by prolonged recovery from desensitization [24], [32]. To measure the recovery of ASICs from desensitization, we evoked ASIC currents in nucleated patches from NG2 cells by applying a double-pulse protocol (Fig. 4*C*). The ratio of amplitudes of second and first responses was plotted against the time interval. Data were fit with a single-exponential function, yielding a recovery time constant (τ) of 16 s (Fig. 4*D*). The time course is comparable to that of homomeric ASIC1a channels in BCs (red curve) but not that of heteromeric ASIC1a/2 channels in O-LM cells (blue curve) (data reproduced from [24]). Taken together, the sensitivity to PcTX1 and the gating properties, particularly the profound tachyphylaxis with slow recovery from desensitization found here, are reminiscent of homomeric ASIC1a channels [24], [32].

### ASIC1a channel activation induces [Ca^2+^]_i_ elevation

**Figure 5:**
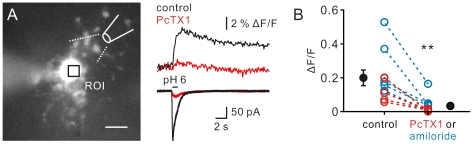
Activation of ASIC1a channels results in [Ca^2+^]_i_ elevation. (A) Fluorescence image of an NG2 cell loaded with OGB-1. Square, the region of interest (ROI). Scale bar, 10 µm. Traces, an example of focal acid puffing to an NG2 cell induced changes of fluorescence signals (top) at the ROI in the control (black) and in the presence of PcTX1 (red) and their corresponding inward currents (bottom). (B) Summary of the change of peak amplitude of [Ca^2+^]_i_ in the control and in the presence of PcTX1 or amiloride. **P<0.005.

Activation of ASIC1a homomers is known to increase intracellular Ca^2+^ [17], [18]. We thus examined whether activation of ASIC channels leads to [Ca^2+^]_i_ elevation in NG2 cells (Fig. 5*A*). To measure [Ca^2+^]_i_ changes, we loaded the cell with a Ca^2+^ indicator OGB-1 (20 µM) via the whole-cell recording pipette. After loading for at least 15 min, focal acid puff to the NG2 cell (*V*-clamped at −80 mV) in the presence of TTX (1 µM), gabazine (1 µM) and kynurenic acid (2 mM) induced an ASIC current (black trace, 308±72 pA, *n* = 10; the representative traces in Fig. 5*A* bottom) with a concomitant transient increase of [Ca^2+^]_i_ (black trace, peak Δ*F*/*F*  = 0.20±0.05, *n* = 10; the representative traces in Fig. 5*A* top). Bath-application of either PcTX1 (30 nM, Fig. 5*A*) or amiloride (100 µM) markedly inhibited the ASIC current (red trace, 32±7 pA, Fig. 5*A* bottom) as well as the [Ca^2+^]_i_ transient (red trace, peak Δ*F*/*F*  = 0.03±0.02, Fig. 5*A* top) (*n* = 10; *P*<0.005, Wilcoxon signed-rank test; Fig. 5*B*).

### Hippocampal NG2 glia have ASIC1a immunoreactivity

**Figure 6:**
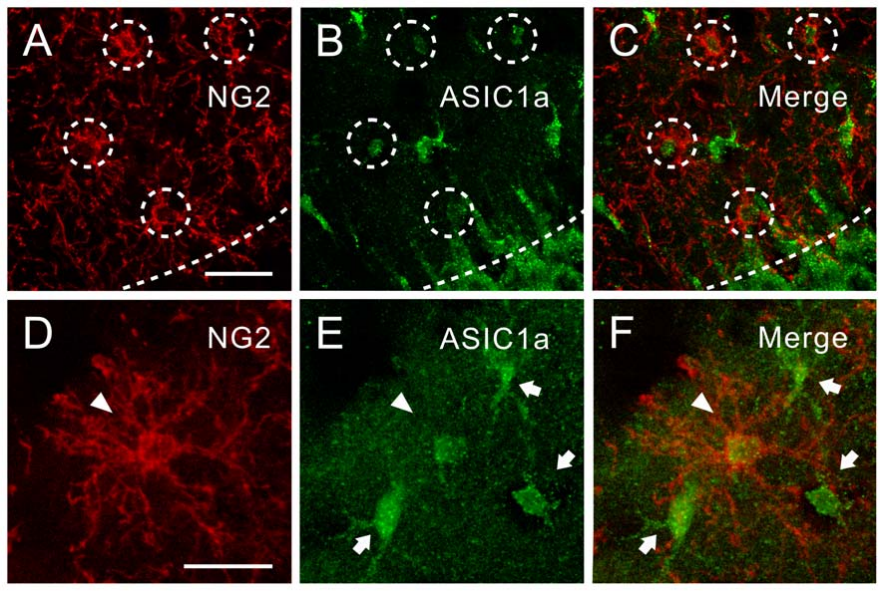
Hippocampal NG2 cells express ASIC1a subunit proteins. (A) An example of images showing NG2 immunoreactivity of CA1 *stratum radiatum*. Dashed circles denote NG2 immunoreactive cells. Dashed lines demarcate the border between *stratum radiatum* and *stratum pyramidale*. Scale bar, 30 µm. (B) The same slice showing ASIC1a immunoreactivity of CA1 *stratum radiatum*. (C) Images of (A) and (B) are superimposed. (D) A magnified NG2-immunoreactive cell (arrowhead) in the *stratum radiatum*. Scale bar, 20 µm. (E) Immunostaining of the same cell (arrowhead) for ASIC1a subunit protein. Arrows, NG2-negative but ASIC1a-positive cells. (F) Images of (D) and (E) are superimposed.

Finally, to investigate whether NG2 cells express ASIC1a channel proteins, we double immunolabeled hippocampal sections with antibodies specific for NG2 (Fig. 6*A*) and ASIC1a subunit proteins (Fig. 6*B*). NG2 immunoreactivity was seen throughout the hippocampus. An example of images showed that ASIC1a staining (Fig. 6*B*) was observed in most NG2-stained cells in the CA1 *stratum radiatum* (Fig. 6*C*). A magnified image of an NG2 cell in the *stratum radiatum* (Fig. 6*D*) showed that ASIC1a (Fig. 6*E*) and NG2 were co-localized (Fig. 6*F*). Overall, 81±3% (*n* = 9 sections, 4 animals) of NG2-immunoreactive cells were ASIC1a positive. Notably, judging from the size of the cells and their location within the hippocampus, some putative interneurons (a small number of scattered cells; arrows in Fig. 6*E*) were intensely immunoreactive for ASIC1a, consistent with our finding and others that ASIC channels are abundantly expressed in GABAergic interneurons [20], [24].

## Discussion

We have reported here that NG2-expressing cells exhibit the proton-activated current that resembles those mediated by recombinant ASICs expressed in heterologous systems [30], [32] and native ASICs in many different types of hippocampal neurons [24]. The pH dependence of these ASIC currents in NG2 cells is comparable to that of native ASICs found in central neurons [24]. Their desensitization time constant (<1 s) is comparable to those of native neuronal ASICs and their reversal potential is close to the *E*_Na_ [24], [32]. Finally, it is blocked by the DEG/ENaC channel blocker amiloride.

The following pieces of evidence further suggest that the Ca^2+^-permeable ASIC1a homomer is the predominant component in NG2 cells. First, the ASIC current in NG cells recovers slowly (τ = 16 s) from desensitization, reminiscent of the prolonged recovery property of heterologously expressed ASIC1a homomers (∼7 s) [32] and native ASIC1a homomers in fast-spiking interneurons (∼43 s) [24]. Second, the ASIC current is largely (>75%) blocked by PcTX1, a specific blocker for ASIC1a homomers [34], [35]. Third, activation of ASICs increases [Ca^2+^]_i_ in NG2 cells [17], [18]. Finally, immunocytochemistry shows that NG2(+) cells are ASIC1a immunoreactive.

Nevertheless, it is yet unknown why the Hill coefficient (0.9) of the ASIC activation curve in NG2 cells is relatively low compared to the reported value (1.64) for recombinant ASIC1a homomers expressed in *Xenopus* oocytes [33]. There are several possible explanations for that. First, it is possible that the ASIC currents in NG2 cells is a mixture of various ASIC channels (the Hill coefficient of heteromeric ASIC1a/2 is lower than that of homomeric ASIC1a) [33]. Indeed, 24% of ASICs is resistant to PcTX1 in the present study. We also made a comparison with previous studies of native ASICs recorded from various neurons. In cultured rat inferior colliculus neurons, the Hill coefficient is 0.92 and the 50% of total ASIC currents is PcTX1-resistant [36]. In cultured cortical neurons, the Hill coefficient is around 1.0 [17], [37] (Xiong, personal communication) and the 50% of total current is PcTX1-resistant component. Therefore, the low Hill slope factor suggests a mixture of various ASIC channels or other non-ASIC channels in NG2 cells. Second, it is also possible that properties of native ASICs may differ considerably in various expression systems. Finally, the dose-response curve was obtained with a series of acidic (from high to low pH) solution tests. Given that the homomeric ASIC1a channel is featured by the profound tachyphylaxis with slow recovery from desensitization, the ASIC currents activated by lower pH solutions may suffer from prior desensitization and thus the responses were likely underestimated.

Intriguingly, the expression of ASIC channels in NG2 cells appears to be quite different from several types of hippocampal neurons. As compared with neuronal ASIC expression, NG2 cells express ASIC1a channels at very high density. The ASIC current density of nucleated patch from NG2 is at least 6-fold larger than those from CA1 PNs, dentate gyrus GCs, and fast-spiking BCs [24]. Although the ASIC current density of O-LM cells appears to be comparable to that of NG2 cells, the subunit composition of ASICs in O-LM cells is quite different. The majority of ASICs in O-LM cells is Ca^2+^-impermeable ASIC1a/2 heteromers rather than Ca^2+^-permeable ASIC1a homomers [24].

Several mechanisms underlie Ca^2+^ signaling in NG2 cells. Depolarizing GABA was shown to trigger Ca^2+^ influx in NG2 cells via Na^+^/Ca^2+^ exchangers [38]. Glutamate release from neurons evokes AMPA- and NMDA-receptor-mediated Ca^2+^ signaling, whereas ATP released by astrocytes also induces [Ca^2+^]_i_ elevation via P2Y_1_ and P2X_7_ purine receptors in NG2 cells [5], [6]. In the present study, acid puff-elicited [Ca^2+^]_i_ elevation in NG2 cells was unlikely through synaptic and neuronal network activity because ionotropic glutamate and GABA_A_ receptor blockers and TTX, a voltage-gated Na^+^ channel blocker, were present throughout the experiments. Most notably, Ca^2+^ transient changes were largely prevented by bath application of PcTX1 or amiloride.

ASICs play a vital role in sensing environmental pH changes. In the heart, ASIC3 channels sense modest pH changes that occur during angina [39]. In the CNS, activation of neuronal ASICs by extracellular pH reductions during epileptic seizures causes enhancement of inhibitory tone, thereby suppressing over-excited cortical networks [20]. A more recent study showed that ASIC2 channels are likely to be the pressure sensor molecule of baroreceptors [40]. Yet little is known about the physiological functions subserved by ASIC1a channels in NG2 cells. NG2 cells, a major source of remyelinating oligodendrocytes, play a critical role in myelination during postnatal brain maturation [9], [41]. The myelination process is thought to be dependent on neuronal activity [42]. Extracellular pH shifts can occur within milliseconds of neural activity, including synaptic transmission and high-frequency action potential discharge [43], [44]. ASIC1a channel is thus suited as an activity-dependent transducer, which converts neuronal activity into intracellular Ca^2+^ signaling and thereby controls NG2 cell division and differentiation.

NG2 cells proliferate and/or release a variety of neuroprotective factors in response to pathological changes [3], [14], [45]. In demyelinating diseases, NG2 cells are recruited to lesions (for review, see [41]). After focal ischemia, GABA concentration drastically increases at the injured regions [46], [47]. GABA_A_ receptor activation induces NG2 cell migration and BDNF release through activation of a Na^+^ channel-Na^+^/Ca^2+^ exchanger pathway [3], [38]. In many pathological conditions, including ischemia, epilepsy and multiple sclerosis, tissue acidosis is a common feature and contributes to brain injury [17]–[20]. By analogy with the depolarizing GABA, activation of ASIC1a channels in NG2 cells by protons might cause membrane depolarization, thereby contributing to the production of BDNF and other neurotrophic factors through Ca^2+^-dependent mechanisms [3]. Moreover, direct Ca^2+^ influx through ASIC1a homomers might also contribute to NG2 cell proliferation or migration after seizures or ischemia [45]. An important future challenge is to define cellular events subsequent to the [Ca^2+^]_i_ elevation in NG2 cells, including immediate effects such as triggering secretion of trophic factors as well as long-term changes in gene expression and cell proliferation or differentiation.

### Caveat

In the present study, we have rigorously tested SR101-negative, biocytin-filled cells for *post-hoc* histology and find that all (42 of 42 cells) of them are NG2-immunoreactive and do not exhibit dye coupling through gap junctions. Nevertheless, our approach may still suffer from the caveats associated with NG2 cell identification. SR101-negative cells may include “complex” astrocytes, which have similar electrophysiological properties of NG2 cells [23], [27] (on the other hand, it is possible that the “complex” astrocytes described by previous studies may also include a population of NG2 cells, see Discussion in [23]). In nucleated patch recordings, the success of morphological reconstruction from biocytin-filled cells was relatively low, thereby hampering the identification of cell morphology and NG2 immunoreactivity. Moreover, NG2 cells consist of a heterogeneous population of cells. Based on our criteria, we may obtain a biased sample of the NG2 cell population and failed to identify NG2 cells in discrete states of differentiation. Thus, caution is needed in interpreting the results. A future study using transgenic mice would allow an unbiased sampling of this population and unambiguous identification of cells in discrete states of differentiation [48].
